# Myopic choroidal neovascularization with dilated choroid vessels is prone to progression into subretinal fibrosis following anti-vascular endothelial growth factor therapy: a retrospective study

**DOI:** 10.1186/s40662-025-00450-4

**Published:** 2025-08-10

**Authors:** Xiangjun She, Qiwei Cai, Wangjing Yao, Shixin Zhao, Zhe Lv, Suyan Shan, Jiwei Tao, Yun Zhang

**Affiliations:** 1 National Clinical Research Center for Ocular Diseases, Eye Hospital, Wenzhou Medical University, Wenzhou, 325027 China; 2 Taizhou Hospital of Zhejiang Province Affiliated to Wenzhou Medical University, Taizhou, 318000 China; 3 The Second Hospital of Jiaxing, Jiaxing, 314000 China

**Keywords:** Subretinal fibrosis, Choroidal neovascularization, Anti-VEGF therapy, Optical coherence tomography

## Abstract

**Background:**

This retrospective study aimed to identify risk factors for subretinal fibrosis (SF) and evaluate the response to anti-vascular endothelial growth factor (anti-VEGF) therapy in patients with myopic choroidal neovascularization (mCNV), with a specific focus on the role of dilated choroidal vessels (DCVs) in disease progression.

**Methods:**

In this retrospective study, patients with high myopia (spherical equivalent < −6.0 D, pathological myopia, Asian ethnicity) and active mCNV lesions, diagnosed between 2021 to 2023, were evaluated. The location of DCVs and mCNV was assessed, and macular thickness, submacular choroid thickness, best-corrected visual acuity, CNV area, and flow density were measured at baseline and during follow-up. The presence of posterior staphyloma was evaluated at baseline. SF around the mCNV was evaluated lesions during follow-up. The time to SF detection was recorded using survival analysis. Risk factors for SF were analyzed using Kaplan–Meier and multivariable Cox regression analyses.

**Results:**

A total of 46 eyes from 46 patients were included, with a mean age of 54.17 ± 14.37 years, and a baseline spherical equivalent of 12.36 ± 3.21 D. The logarithm of the minimum angle of resolution for the mean visual acuity was 0.70 (0.40–1.30), and the mean macular thickness was 313.11 ± 63.57 μm at baseline. DCV was detected in 29 of the 46 eyes (63.0%), and the median time to detect SF was 43.41 [95% confidence interval (CI): 37.27–49.55] months. Multivariable Cox regression analysis identified submacular DCV [hazard ratio (HR): 14.93, 95% CI: 5.72–38.91, *P* < 0.001) and absence of posterior staphyloma (HR: 43.48, 95% CI: 12.15–156.32, *P* = 0.002) as independent predictors of SF. The presence of DCV under the fovea compared to the peripheral zone achieved a poorer therapeutic response and was prone to progress to SF after anti-VEGF therapy (*P* = 0.041).

**Conclusions:**

Submacular DCV is associated with poor therapeutic response to anti-VEGF therapy and an increased risk of SF in patients with mCNV.

## Background

Pathological myopia (PM) is a leading cause of visual impairment and blindness worldwide, especially in Asian countries [1, 2]. Among its most severe complications, Myopic choroidal neovascularization (mCNV) stands out as a primary contributor to vision loss in patients with PM [3], with mCNV developing in approximately 5%–11% of affected eyes [4] Its presence caused serious visual morbidity [5].

At present, intravitreal anti-vascular endothelial growth factor (anti-VEGF) therapy is the first-line treatment for mCNV [6, 7]. Anti-VEGF agents, either with ranibizumab or bevacizumab could reduce vascular permeability by inhibiting VEGF, thereby leading to lesion regression and improvement in visual acuity [4]. However, despite initial visual gains, many patients exhibit suboptimal responses and gradual vision loss over time [8]. One key factor contributing to this poor long-term outcome is the development of subretinal fibrosis (SF), which occurs in approximately 40.7% of eyes following anti-VEGF therapy. Although, anti-VEGF treatment stabilized the lesion and improvement of visual acuity. SF is considered as the main reason for vision loss after treatment [9].

From neovascularization membrane to a fibrovascular lesion, which ultimately results in fibrotic scar formation, disruption of photoreceptors and the retinal pigment epithelium (RPE), leading to permanent damage and a poor visual outcome [10]. To date, the risk factors for SF remain unclear [11]. Previous studies have indicated that being less than 45 years old and having a best-corrected visual acuity (BCVA) of less than 60 letters at baseline are predictive factors of fibrosis [12]. The anatomy studies of CNV and choroid with SF in the context of dilated choroidal veins were lacking.

In a recent study, we observed that dilated and tortuous choroidal veins often appeared adjacent to mCNV lesions, and dilated choroidal vessels (DCVs) were commonly detected in eyes with PM [13]. Therefore, this study aimed to further investigate whether and how the morphological structure of mCNV affects regression and treatment in patients with DCVs.

## Methods

### Patient selection

This retrospective study was conducted at the Affiliated Eye Hospital of Wenzhou Medical University, Wenzhou, China. This study was approved by the Ethics Committee of Wenzhou Medical University Eye Hospital, Wenzhou, Zhejiang Province, China (H2022-010-K-10-01) and conducted in accordance with the principles outlined in the Declaration of Helsinki. We reviewed the medical records of patients who visited the hospital from January 2021 to December 2023. All enrolled patients provided written informed consent before the procedure and received a 1 + pro re nata (PRN) regimen of ranibizumab (0.5 mg) or conbercept (0.5 mg). During subsequent follow-up visits, if mCNV activity had completely subsided, no further injections were given unless recurrence was observed. If mCNV activity persisted, however, monthly injections continued until the lesion stabilized [14]. The inclusion criteria were: (1) refractive error (spherical equivalent) ≤ −6.00 D or axial length exceeding 26.00 mm with concomitant scleral, choroidal, and retinal degenerative changes; (2) fundus photography, optical coherence tomography (OCT), OCT angiography (OCTA), or fluorescein angiography (FA) suggestive of the presence of naïve active CNV; and (3) a minimum follow-up period of 12 months. Given the intrinsic relationship between an individual’s two eyes, if both met the inclusion criteria, one eye was randomly selected for the study. The exclusion criteria included: (1) CNV in the scarring or atrophic stage at the time of initial diagnosis; (2) CNV secondary to other diseases, such as punctate inner chorioretinopathy, multifocal choroiditis, or uveitis; (3) intraocular surgery in addition to anti-VEGF treatment during follow-up; (4) severe posterior segment complications, such as retinal detachment or macular hole; (5) previous anti-VEGF treatment at another institution; (6) CNV area on the OCTA image exceeding 3 × 3 mm^2^ or CNV area too large to fit within the 3 × 3 mm^2^ area; and (7) No major follow-up visits (≥ 2) were missed.

### Data collection

The participants’ age, sex, and initial BCVA were recorded, with BCVA results converted to the logarithm of the minimum angle of resolution (logMAR) for analysis. Refractive errors were obtained from medical records during the initial examination. All patients underwent indirect ophthalmoscopy, color fundus photography (TRC-50DX, Topcon Corporation, Tokyo, Japan) or confocal scanning laser ophthalmoscopy (cSLO; Optos Daytona, Optos, England), structural spectral-domain OCT (SD-OCT) (Spectralis SD-OCT; Heidelberg Engineering, Germany), OCTA (Optovue, Fremont, America) or fundus fluorescein angiography (FFA)/indocyanine green angiography (ICGA) (HRA Spectralis, Heidelberg, Germany). BCVA, OCTA, and OCT were recorded before treatment and at 1, 3, 6 and 12 months post-treatment. Central macular thickness (CMT), central foveal thickness (CFT), subfoveal choroidal thickness (SFCT), CNV area, and CNV flow area were also measured [15].

**Fig. 1:**
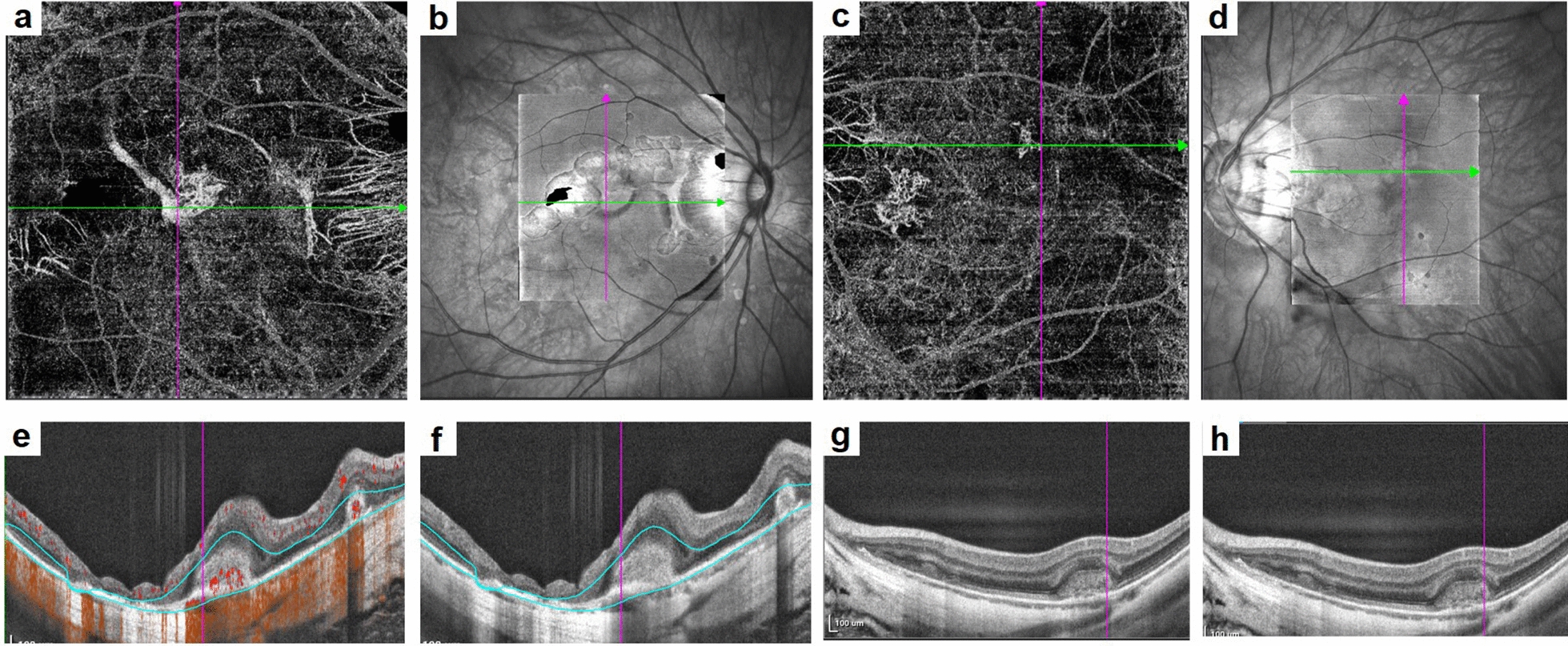
Images of patients with myopic choroidal neovascularization (mCNV), with or without dilated choroidal vessels (DCVs). **a**, **b**, **e**, **f** A female 45-year-old patient with mCNV and a baseline visual acuity of decimal 0.5. **a**, **b** The mCNV structure in the outer retina and a DCV in the choriocapillaris observed as a dilated choroid structure in the cross line in en face optical coherence tomography (OCT). **e**, **f** Horizontal and vertical scans using swept-source OCT (SS-OCT), revealing a hyperreflective structure in the subretinal space. The DCV was visible under the mCNV lesion. **c**, **d**, **g**, **h** A 32-year-old female patient with mCNV, with a visual acuity of decimal 0.6. **c**, **d** The mCNV lesion in the cross-sectional view. **g**, **h** A hyperreflective subretinal lesion without choroidal dilation

All OCT images were evaluated by two independent observers (YZ and XJS). Fibrosis was defined as white or yellow subretinal tissue on fundus photographs and as homogeneous, highly hyperreflective lesions with relatively well-defined borders on OCT images, often accompanied by atrophy and thinning of the surrounding outer retina [16]. DCV was defined as a large choroidal vein with a diameter greater than or equal to at least twice the diameter of the adjacent vein on ICGA and OCTA [17] (Fig. 1), they were classified into absence, submacular and peripheral groups according to the position of the DCV in relation to the macula. The CNV area (contour area) and CNV blood flow area selected by the frame were automatically calculated using built-in software along the CNV edge contour. Measurements were performed independently by two observers. If the discrepancy exceeded 10%, a reevaluation was conducted [18, 19]. CNV was classified as subfoveal or parafoveal based on whether it was within 1 mm of the foveal center. Lesions located outside the macula were classified as peripheral CNV. The presence or absence of posterior staphyloma was reviewed based on pigmentary changes typical of staphyloma borders on 200° ultra-widefield fundus images Optos [20].

### Statistical analysis

Statistical analyses were conducted using commercial software (SPSS for Windows, version 26.0.0; SPSS Inc., Chicago, IL, USA). Snellen visual acuity was converted to logMAR values for statistical purposes. The Kolmogorov–Smirnov test was used to determine the normality of all continuous variables. Normally distributed data are expressed as mean ± standard deviation; non-normally distributed data are expressed as median (interquartile range). Independent t-test or the Mann–Whitney U test was used for comparisons of normally distributed or non-normally distributed continuous variables, respectively, and the Pearson's χ^2^ test was used for comparison of categorical variables. The Kaplan–Meier (KM) survival analysis and Cox proportional risk modeling were performed for the time-to-fibrosis events. Repeated-measures analysis of variance was used to compare changes at each time point. Statistical significance was set at a two-tailed *P* value < 0.05. The Bonferroni method was used to correct for multiple comparisons.

## Results

### Demographics and clinical characteristics

The demographics of all participants are summarized in Table 1. A total of 46 eyes received a 1 + PRN regimen. We included 23 patients treated with ranibizumab and 23 patients treated with conbercept. The mean age was 54.17 ± 14.37 years. The median number of injections was 3.00 [interquartile range (IQR), 2.00–4.00]. No significant differences were observed in the mean number of injections or visual acuity changes between the treatment groups. KM survival analysis revealed a median time to fibrosis onset of 43.41 [95% confidence interval (CI), 37.27–49.55] months. Overall, no significant differences in baseline BCVA, age, drug type, CNV location, CMT, CFT, SFCT, or presence of DCV were observed in patients with or without SF. Eyes with SF exhibited significantly larger CNV lesion area [median: 0.64 mm^2^ (IQR: 0.36–1.92) vs. 0.31 mm^2^ (0.12–0.66), *P* = 0.009] and CNV flow area [0.53 mm^2^ (0.30–1.39)] vs. 0.25 mm^2^ (0.09–0.56), *P* = 0.011] compared to non-SF eyes. In contrast, posterior staphyloma was more frequently observed in the non-SF group [32 (78.0%) vs. 9 (22.0%), *P* = 0.002].

**Table 1:** Demographic and clinical characteristics at baseline

Parameters	Total eyes (n = 46)	SF (n = 14)	Without SF (n = 32)	*P* value
Sex, female (%)	78.3%	85.7%	75.0%	0.673
SE (D)	−12.36 ± 3.21	−12.87 ± 2.43	−12.14 ± 3.51	0.485
BCVA (logMAR)	0.70 (0.40–1.30)	0.75 (0.65–1.30)	0.52 (0.33–1.30)	0.163
Age (years)	54.17 ± 14.37	55.50 ± 16.37	53.59 ± 13.3	0.684
Drug given	Ranibizumab/Conbercept	7/7	16/16	1.000
CNV location	Subfoveal 20 (43.5%) Parafoveal 8 (17.4%) Peripheral 18 (39.1%)	8 (57.1%) 3 (21.4%) 3 (21.4%)	12 (37.5%) 5 (15.6%) 15 (46.9%)	0.246
CMT (μm)	313.11 ± 63.57	303.21 ± 56.53	317.44 ± 66.80	0.491
CFT (μm)	307.00 (224.00–395.50)	284.50 (206.75–333.25)	357.00 (224.00–410.00)	0.316
SFCT (μm)	52.50 ± 25.71	60.31 ± 28.89	50.25 ± 23.29	0.375
CNV area (mm^2^)	0.36 (0.19–0.91)	0.64 (0.36–1.92)	0.31 (0.12–0.66)	0.009*
CNV flow area (mm^2^)	0.31 (0.14–0.66)	0.53 (0.30–1.39)	0.25 (0.09–0.56)	0.011*
DCV (presence)	29 (63.0%)	9 (64.3%)	20 (62.5%)	0.908
DCV location				
	Absence (37.0%) Subfoveal (34.8%)	5 8	12 8	0.035*
	Peripheral (28.2%)	1	12	
Posterior staphyloma	41 (89.1%)	9 (64.3%)	32 (100.0%)	0.002*

*SF* = subretinal fibrosis; *SE* = spherical equivalent; *BCVA* = best-corrected vision acuity; *CNV* = choroidal neovascularization; *CMT* = central macular thickness; *CFT* = central foveal thickness; *SFCT* = subfoveal choroidal thickness; *DCV* = dilated choroid vessel

* denotes *P* < 0.05, which is considered as statistically significant

### Retinal and choroid structure changes in patients with mCNV with or without SF

Retinal thickness, BCVA, and choroidal thickness were used to assess therapeutic response in patients with or without SF. Changes in BCVA (*P* = 0.065–0.221), CFT (*P* = 0.259–0.802), and SFCT (*P* = 0.213–0.375) were not significantly different between patients with and without SF (Table 2). However, the CNV area and flow area were significantly larger in patients with SF than those without SF at 3 months [0.63 (0.34–0.80) mm^2 ^vs. 0.23 (0.06–0.59) mm^2^, *P* = 0.041; 0.41 (0.24–0.65) mm^2^ vs. 0.20 (0.05–0.45) mm^2^, *P* = 0.036, respectively] and 12 months [0.61 (0.28–1.30) mm^2^ vs. 0.24 (0.07–0.58) mm^2^, *P* = 0.020; 0.61 (0.28–1.30) mm^2^ vs.0.24 (0.18–0.45) mm^2^, *P* = 0.011, respectively] after therapy (Table 2). However, after Bonferroni correction, only the changes of CNV flow area remain statistically significant.

**Table 2:** Visual and retinal-choroidal structures in eyes with mCNV with or without SF

Parameters	Total eyes	SF	Without SF	*P* value
BCVA (logMAR)				
Baseline BCVA	0.70 (0.40–1.30)	0.75 (0.65–1.08)	0.52 (0.33–1.30)	0.163
BCVA 3 months	0.30 (0.15–0.57)	0.52 (0.22–1.00)	0.30 (0.15–0.49)	0.221
BCVA 6 months	0.30 (0.14–0.57)	0.52 (0.21–0.81)	0.26 (0.10–0.49)	0.065
BCVA 12 months	0.30 (0.15–0.70)	0.46 (0.22–0.85)	0.30 (0.11–0.40)	0.105
CFT (μm)				
Baseline CFT	307.00 (224.00–395.50)	284.50 (206.75–333.25)	357.00 (224.00–410.00)	0.316
CFT 3 months	224.00 (186.50–307.00)	224.500 (187.00–290.00)	218.00 (186.50–330.00)	0.802
CFT 6 months	218.00 (189.00–288.50)	221.50 (186.00–260.75)	211.00 (191.50–309.00)	0.652
CFT 12 months	219.00 (181.00–297.00)	233.50 (184.00–292.50)	207.00 (181.00–297.50)	0.259
SFCT (μm)				
Baseline SFCT	52.50 ± 25.71	57.64 ± 30.89	50.25 ± 23.29	0.375
SFCT 3 months	51.37 ± 23.15	58.92 ± 28.26	48.24 ± 20.44	0.213
SFCT 6 months	50.80 ± 22.68	58.42 ± 25.52	47.66 ± 21.06	0.241
SFCT 12 months	49.41 ± 21.23	56.58 ± 27.56	46.45 ± 17.73	0.273
CNV area (mm^2^)				
Baseline area	0.36 (0.19–0.91)	0.64 (0.36–1.92)	0.31 (0.12–0.66)	0.009*^#^
Area 3 months	0.31 (0.09–0.67)	0.63 (0.34–0.80)	0.23 (0.06–0.59)	0.041*
Area 6 months	0.34 (0.07–0.68)	0.71 (0.34–1.23)	0.23 (0.07–0.59)	0.056
Area 12 months	0.36 (0.16–0.77)	0.61 (0.28–1.30)	0.24 (0.07–0.58)	0.020*
CNV flow area (mm^2^)				
Baseline flow area	0.31 (0.14–0.66)	0.53 (0.30–1.39)	0.25 (0.09–0.56)	0.011*
Flow area 3 months	0.24 (0.06–0.53)	0.41 (0.24–0.65)	0.20 (0.05–0.45)	0.036*^#^
Flow area 6 months	0.26 (0.06–0.54)	0.57 (0.24–0.87)	0.19 (0.05–0.49)	0.049*
Flow area 12 months	0.41 (0.25–0.79)	0.61 (0.28–1.30)	0.24 (0.18–0.45)	0.011*^#^

*mCNV* = myopic choroidal neovascularization; *SF* = subretinal fibrosis; *BCVA* = best-corrected vision acuity; *CFT* = central foveal thickness; *SFCT* = subfoveal choroidal thickness; *CNV* = choroidal neovascularization

* denotes *P* < 0.05, which is considered statistically significant

^#^ indicates statistically significance after Bonferroni correction

### Survival modeling analysis of factors influencing SF

The KM analysis was used to identify the risk factors for SF. This analysis indicated that the positions of the DCV significantly influenced the probability of fibrosis occurrence (Breslow χ^2^ = 12.119, *P* = 0.007; Fig. 3). Patients with posterior staphyloma are less likely to develop fibrosis compared to non-staphyloma cases (Breslow χ^2^ = 7.994, *P* = 0.005). Additionally, those with a CNV area ≥ 0.363 mm^2^ are prone to develop fibrosis much earlier (Breslow χ^2^ = 4.121, *P* = 0.042), and patients with a BCVA worse than 0.699 logMAR were at a higher risk of developing fibrosis (Breslow χ^2^ = 4.489, *P* = 0.034; Table 3).

**Table 3:** The risk factors to predict SF in mCNV patients

Variables	Subgroup	Univariate analysis	Multivariate analysis			
			*P* value	HR	95% CI for HR	
					5%	95%
DCV location	Absence (39.9%)					
	Submacular (34.8%)	0.007	0.039	14.93	1.14	197.45
	Peripheral (28.3%)					
Posterior staphyloma	Absence (10.9%)	0.005	0.002	43.48	4.22	446.65
	Presence (89.1%)					
CNV area	< 0.363 mm^2^ (50.0%)	0.042				
	≥ 0. 363 mm^2^ (50.0%)					
BCVA	< 0.699 logMAR (45.7%)	0.034				
	≥ 0.699 logMAR (54.3%)					

*mCNV* = myopic choroidal neovascularization; *DCV* = dilated choroidal vessel; *CNV* = choroidal neovascularization; *BCVA* = best-corrected visual acuity; *CI* = confidence interval; *HR* = hazard ratio

### Therapeutic response in patients with mCNV having DCV at different locations

**Fig. 2:**
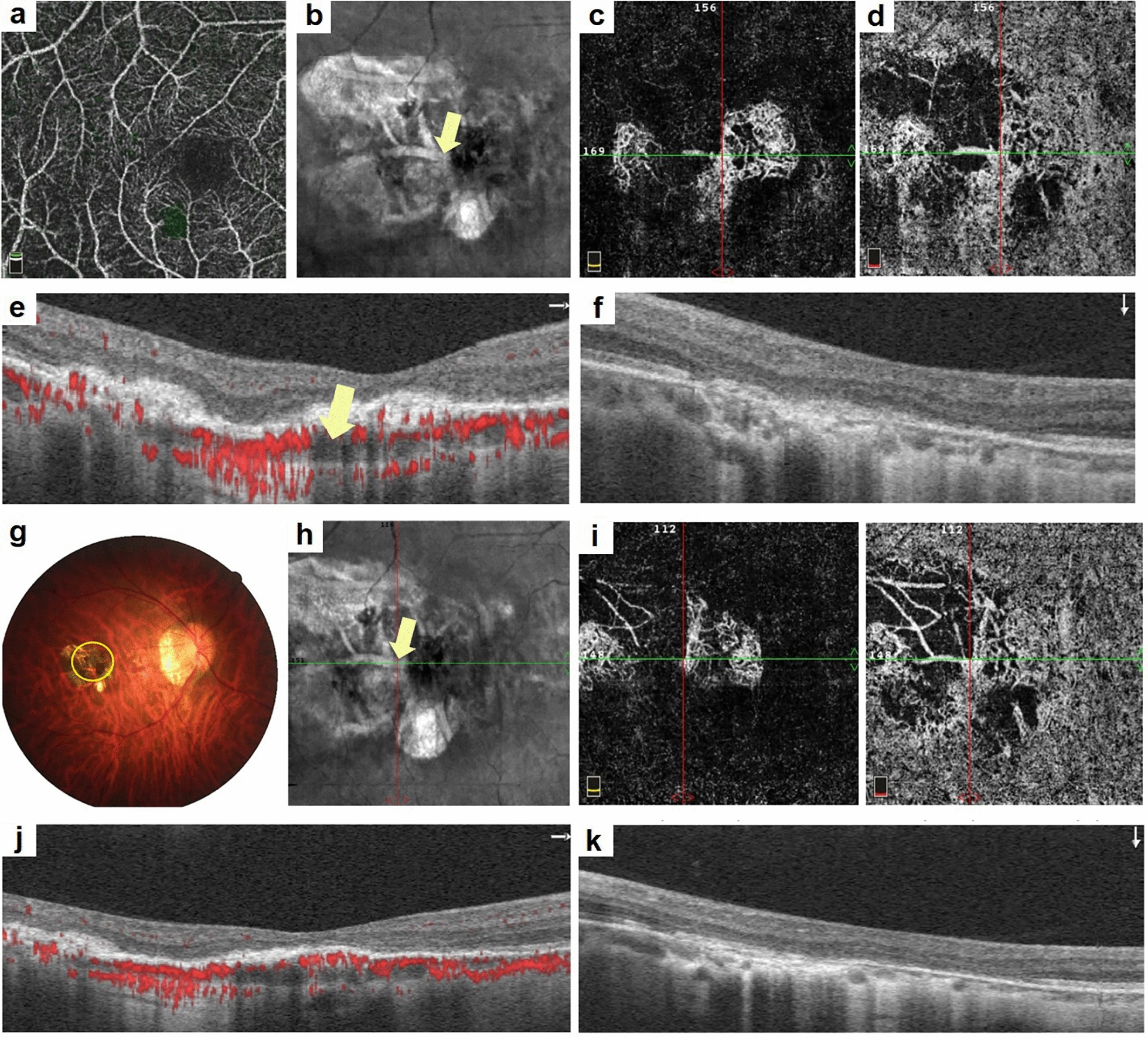
Progression of subretinal fibrosis (SF) in a patient with myopic choroidal neovascularization (mCNV) with dilated choroidal vessel (DCV) after anti-vascular endothelial growth factor (anti-VEGF) therapy. A 32-year-old female patient was diagnosed with mCNV. **a** The superior retinal structure. **b** The structure with DCV by en face optical coherence tomography (OCT), the yellow arrow depicts the DCV. **c** The mCNV lesion in the outer retina structure is shown while (**d**) highlights the lesion at the choriocapillaris level, with the CNV located around the DCV. **e**, **f** The B-scan by horizontal and vertical structure using OCT, where a hyperreflective deposit is detected in the macular region with a dilated choroid structure, marked by the yellow arrow. After 1 month of anti-VEGF therapy, SF is detected in (**g**, yellow circle), while (**h**) the DCV remains unchanged. **i** The mCNV lesion decreased in the outer retina structure and in choriocapillaris level. **k** The mCNV lesion appears smaller than at baseline from the B-scan, with a subretinal deposit reduced compared to baseline but still present in the subretinal macular region, characterized by an interrupted retinal pigment epithelium (RPE) structure in (**j**) and (**k**)

As submacular DCV is a known risk factor for SF formation, we compared therapeutic responses between the submacular and peripheral DCV groups. As the CFT, CNV area, and flow area were not normally distributed, they were transformed into normally distributed data using an open square root transformation. These results indicated that mCNV patients with submacular DCVs had a greater proportion of fibrosis than those with peripheral DCVs (Fig. 2). The SFCT was greater in patients with submacular DCV at 3 months (63.38 ± 28.01 μm vs. 39.33 ± 17.49 μm, *P* = 0.035) and 6 months (62.75 ± 27.32 μm vs. 36.67 ± 19.18 μm, *P* = 0.034). CNV areas were also larger in patients with submacular DCVs at 6 months [0.71 (0.31, 1.15) mm^2^ vs. 0.19 (0.00, 0.55) mm^2^, *P* = 0.041]. The CNV flow areas were also larger in patients with submacular DCVs at 6 months [0.55 (0.27, 0.83) mm^2^ vs. 0.16 (0.00, 0.47) mm^2^, *P* = 0.022) and at 12 months [0.49 (0.28, 0.78) vs. 0.15 (0.00, 0.55), *P* = 0.045]. The differences remained significant after Bonferroni correction. No statistical significance with respect to the BCVA or CFT were observed between the two groups (Table 4).

**Table 4:** Demographics, clinical characteristics and outcome in eyes with DCV patients in different locations

Variables	Submacular type	Peripheral type	*P* value
Sex, female (%)	81.25%	69.23%	0.752
Age (years)	57.25 ± 13.31	54.15 ± 15.39	0.566
SE (D)	−12.51 ± 3.19	−14.38 ± 3.22	0.130
BCVA (logMAR)			
Baseline BCVA	0.91 ± 0.40	0.59 ± 0.35	0.797
BCVA 3 months	0.45 ± 0.28	0.43 ± 0.62	0.743
BCVA 6 months	0.49 ± 0.35	0.30 ± 0.39	0.446
BCVA 12 months	0.53 ± 0.43	0.33 ± 0.39	0.308
CFT (μm)			
Baseline CFT	393.50 (277.00, 445.00)	310.00 (211.50, 365.00)	0.412
CFT 3 months	307.00 (228.00, 365.00)	197.00 (185.00, 344.00)	0.402
CFT 6 months	275.00 (225.00, 336.50)	205.00 (185.00, 295.50)	0.450
CFT 12 months	297.00 (192.75, 362.00)	197.00 (181.50, 289.00)	0.674
SFCT (μm)			
Baseline SFCT	67.63 ± 35.13	37.56 ± 20.16	0.055
SFCT 3 months	63.38 ± 28.01	39.33 ± 17.49	0.035*
SFCT 6 months	62.75 ± 27.32	36.67 ± 19.18	0.034*
SFCT 12 months	61.38 ± 26.17	37.56 ± 18.00	0.059
CNV area (mm^2^)			
Baseline area	0.75 (0.32, 1.76)	0.09 (0.32, 0.88)	0.193
Area 3 months	0.59 (0.30, 0.78)	0.24 (0.01, 0.53)	0.157
Area 6 months	0.71 (0.31, 1.15)	0.19 (0.00, 0.55)	0.041*
Area 12 months	0.63 (0.33, 1.20)	0.24 (0.00, 0.65)	0.055
CNV flow area (mm^2^)			
Baseline flow area	0.56 (0.27, 1.28)	0.26 (0.08, 0.66)	0.108
Flow area 3 months	0.36 (0.26, 0.62)	0.21 (0.01, 0.41)	0.054
Flow area 6 months	0.55 (0.27, 0.83)	0.16 (0.00, 0.47)	0.022*
Flow area 12 months	0.49 (0.28, 0.78)	0.15 (0.00, 0.55)	0.045*
Time to develop fibrosis (months)	27.19 ± 16.65	28.46 ± 12.27	0.820
Incidence of SF	5/19	9/17	0.041*

*DCV* = dilated choroidal vessel; *SE* = spherical equivalent; *BCVA* = best-corrected vision acuity; *CFT* = central foveal thickness; *SFCT* = subfoveal choroidal thickness; *CNV* = choroidal neovascularization; *SF* = subretinal fibrosis

* denotes *P* < 0.05, which was considered statistically significant, the *P* values were corrected using the Bonferroni method

## Discussion

Here, mCNV is a leading cause of decreased visual acuity in patients with PM. Most patients with mCNV show improved visual acuity after anti-VEGF therapy. SF is a significant factor contributing to the limited improvement in visual acuity and poor response. In addition, eyes with SF are prone to progression to myopic chorioretinal atrophy. The results of our study indicate that eyes with DCV and mCNV are prone to the formation of SF.

Our study showed that the incidence of SF in patients with treated mCNV was 8.7% at the 1-year follow-up and increased to 30.4% throughout the entire visit. Notably, patients presenting with subfoveal DCV have an 18.8% probability of fibrosis within the first year and a 50% incidence over the entire visit. These results are comparable to those of previous studies e.g., Xiao et al. reported an SF rate of 40.7% [12]. We also found that visual outcomes remained poorer in the SF group compared to the non-SF group even at 6 months post-treatment. Prior studies indicate that baseline risk factors associated with SF development include classic CNV, blocked fluorescence observed on FFA, the presence of foveal subretinal fluid, increased foveal thickness, and subretinal tissue [10]. Classic CNV lesions have been shown to progress to SF due to RPE disruption, facilitating subretinal migration and proliferation of RPE cells, which predisposes lesions to scarring following therapy.

**Fig. 3:**
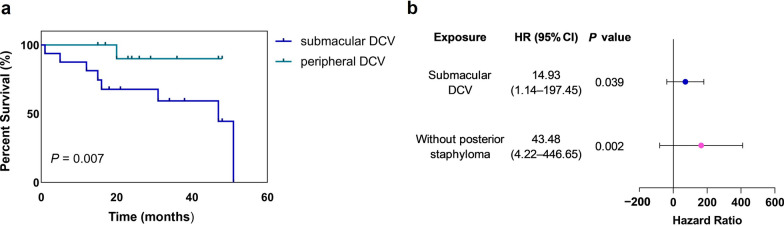
Kaplan–Meier (KM) survival analysis for subretinal fibrosis (SF) after anti-vascular endothelial growth factor (anti-VEGF) therapy. **a** KM survival curves for SF development stratified by dilated choroidal vessel (DCV) location. The subfoveal DCV group exhibited a significantly shorter time to SF onset compared with the peripheral DCV group (median: 37.04 vs. 45.20 months, log-rank *P* = 0.007). **b** Forest plot showing the risk factors with SF by survival model. Both subfoveal DCV and the absence of posterior staphyloma are risk factors for fibrotic outcome in patients with mCNV

We identified DCV as a novel marker for predicting SF in patients with mCNV after therapy. Interestingly, the location of the DCV was related to fibrosis, and thus we further investigated the impact of submacular and peripheral type on fibrosis progression. The survival analysis model revealed that both DCV location and posterior staphyloma are independent predictors of SF. Submacular DCV is a significant risk factor for the development of fibrosis in patients with mCNV (*P* = 0.039), with a risk 14.93 times higher than that in patients with peripheral DCV. The risk of SF occurrence increased to 43.48 times that of those without posterior staphyloma (*P* = 0.002; Fig. 3). Xiao et al. reported that subfoveal mCNV was highly associated with SF [12]. The thicknesses of the SFCT, CNV area and the flow area in the submacular-DCV group were greater than those in the peripheral-DCV group even at baseline and after therapy. The therapy response was also poorer in the submacular DCV group. Data indicated that a dilated choroid was frequently detected in patients with DCV. In our study, a larger CNV size was detected in patients with DCV; it has been reported that a larger CNV size at baseline is a risk factor for SF [11]. This suggests that increased CNV size associated with DCV may contribute to the higher incidence of fibrosis. Furthermore, we observed a shorter mean time to fibrosis onset in the DCV group.

DCVs are commonly detected in 30% of eyes with mCNV [11]. Interestingly, subfoveal DCV was more common in mCNV eyes. Xie et al. showed that DCVs were in contact with mCNVs through a defect in the RPE Bruch’s membrane [17]. DCVs in PM are thought to reflect choroidal ischemia, as proposed by Matsumoto et al. [21] who hypothesized that chronic venous congestion may contribute to the pathogenesis of pachychoroid neovasculopathy [22]. Although the choroid is generally thinner in eyes with PM than healthy eyes, it appeared thicker in the area surrounding DCVs in the mCNV than other regions. Currently, there is no direct evidence available to quantify the ischemic status of DCVs in mCNV-affected eyes. However, a previous study suggested that DCVs reflect ischemic conditions in PM [21]. Furthermore, exudative pachychoroid neovasculopathy accompanied by DCVs has been associated with poorer visual outcomes following anti-VEGF treatment [23]. We hypothesize that choroidal ischemia linked to DCVs may underlie the reduced therapeutic efficacy and increased tendency toward fibrosis observed in patients with mCNV. Angiographic analysis of the blood supply origins in mCNV has revealed that DCVs commonly arise from the short posterior ciliary arteries [24], supporting a potential arterialization connection between mCNVs and DCVs. Anti-VEGF therapies are known to be ineffective against such arterialization. Additionally, in cases of posterior staphyloma, these vessels are displaced peripherally [25–27].

SF often develops within the first year of therapy [28]. Here, the mean time to SF formation was shorter in eyes with submacular DCV than in eyes with peripheral DCV. Anti-VEGF therapy may cause a contraction of the CNV and traction of the surrounding RPE layers, thereby inducing degeneration of the RPE. These factors may be responsible for the poor response in the submacular DCV group after therapy.

Our study presents some limitations that should be considered in the context of CNV therapy. First, the study design was retrospective; a prospective study is needed to confirm our findings. Another shortcoming is that the uneven proportions between the groups result in wide confidence intervals for the survival times. Thus, more precise results can be obtained by increasing the sample size in the future.

## Conclusion

This study indicates that SF is often detected in eyes with DCV and CNV at a rate of approximately 34.4%. Eyes with DCVs in the CNV group, especially those of the subfoveal type, showed a poor therapeutic response and a shorter time to SF development. A thorough examination of the choroid is crucial in guiding therapy for eyes with CNV. Future studies should investigate whether fewer anti-VEGF injections are required to treat this type of CNV in eyes with submacular DCVs*.* Additionally, the development of anti-fibrotic drugs should be explored to improve the visual acuity in these patients.

## Data Availability

The datasets used and analyzed during the current study are available from the corresponding author upon reasonable request.

## Abbreviations

BCVA: Best-corrected visual acuity
CNV: Choroidal neovascularization
CFT: Central foveal thickness
CMT: Central macular thickness
DCV: Dilated choroidal vessel
FFA: Fundus fluorescein angiography
ICGA: Indocyanine green angiography
KM: Kaplan–Meier
mCNV: Myopic choroidal neovascularization
OCTA: Optical coherence tomography angiography
OCT: Optical coherence tomography
PRN: Pro re nata
RPE: Retinal pigment epithelium
SF: Subretinal fibrosis
SFCT: Subfoveal choroidal thickness
SS-OCT: Swept-source optical coherence tomography
VEGF: Vascular endothelial growth factor
anti-VEGF: Anti-vascular endothelial growth factor
